# Aurora kinase A has a significant role as a therapeutic target and clinical biomarker in endometrial cancer

**DOI:** 10.3892/ijo.2015.2842

**Published:** 2015-01-22

**Authors:** KIYOKO UMENE, MEGUMI YANOKURA, KOUJI BANNO, HARUKO IRIE, MASATAKA ADACHI, MIHO IIDA, KANAKO NAKAMURA, YUYA NOGAMI, KENTA MASUDA, YUSUKE KOBAYASHI, EIICHIRO TOMINAGA, DAISUKE AOKI

**Affiliations:** Department of Obstetrics and Gynecology, School of Medicine, Keio University, Tokyo, Japan

**Keywords:** Aurora kinase A, endometrial cancer, chemosensitivity, paclitaxel

## Abstract

Aurora kinase A (AURKA) regulates the cell cycle checkpoint and maintains genomic integrity. AURKA is overexpressed in various malignant tumors and its upregulation induces chromosomal instability, which leads to aneuploidy and cell transformation. To investigate the role of AURKA in endometrial cancer, we evaluated the association of immunohistochemical expression of AURKA with clinicopathological factors. Furthermore, we examined the effects of AURKA inhibition by transfected siRNA in HEC-1B cells on colony-forming ability, invasion and migration capacity, and chemosensitivity. Immunohistochemical staining showed that overexpression of AURKA was significantly associated with tumor grade (P<0.05) and poor histologic differentiation (P<0.05). The recurrence rate also tended to be high in cases with overexpression of AURKA (P<0.1) and these cases also had a tendency for shorter disease-free survival (DFS) (P<0.1). AURKA inhibition in endometrial cancer cell lines significantly decreased cell growth, invasion and migration (P<0.05), and increased chemosensitivity to paclitaxel. We also evaluated the efficacy of a combination of AURKA siRNA and paclitaxel against subcutaneous tumors formed in a nude mouse. After treatment, the tumor volume shrank significantly compared to treatment with paclitaxel only (P<0.05). To our knowledge, this is the first study in endometrial carcinoma to show a correlation between overexpression of AURKA and tumor grade, histological type and sensitivity to paclitaxel. AURKA is a promising therapeutic target in endometrial cancer and the combination therapy with AURKA inhibitors and paclitaxel could be effective for endometrial cancer that is resistant to conventional treatment and has a poor prognosis.

## Introduction

Endometrial cancer is the sixth most common incident cancer in women worldwide, and this prevalence may reflect recent changes in lifestyles of women (1). Endometrial cancer is classified into Types I and II based on etiology and clinical behavior (2,3). Type I tends to appear with age, either before or after menopause, and comprises ~80% of all cases of endometrial cancer. Type I tumors are estrogen-dependent and well-differentiated adenocarcinomas that occur against a background of endometrial hyperplasia and have a favorable prognosis. *PTEN* and *K-ras* gene mutations are connected to their development. Type II tumors are poorly-differentiated adenocarcinomas that tend to occur at a relatively advanced age and include clear cell and serous adenocarcinomas. These tumors are not estrogen-dependent, occur *de novo* against a background of endometrial atrophy, and have a poor prognosis. *P53* gene mutation and high chromosomal stability are connected to their development. Although the clinicopathogenic backgrounds of Types I and II differ, the treatments are similar. Type II adenocarcinomas are resistant to current therapies and these tumors continue to have a poor prognosis. Thus, improved treatment for endometrial cancer requires improved understanding of the carcinogenic mechanism and development of therapeutic strategies that are specific to each patient’s condition.

The human Aurora kinase family includes three subtypes: A, B, and C. Aurora kinase A (AURKA) and B are overexpressed in many human cancer cell-derived cell lines and cancer tissues, and are connected to carcinogenesis (4). AURKA is a G2/M phase serine/threonine kinase that mainly accumulates at centrosomes during late G2 phase anaphase and plays a role in centrosome separation and bipolar spindle formation and stabilization (5,6). AURKA is regulated to ensure proper mitosis, and its overexpression induces an increase in centrosome number and aneuploid cell formation, leading to a significant risk of carcinogenesis (4,7–9). AURKA overexpression occurs in chromosomal region 20q13, at which gene amplification is seen in many human cancers; and is involved in colorectal (10), bladder (11), pancreatic (12), gastric (13) and breast (14) cancers. In ovarian cancer that is a poor prognostic gynecological cancer, AURKA overexpression is also found in cell lines and cancer tissues and is associated with poor prognosis in cancer patients (15,16). AURKA overexpression also increases resistance to taxanes, which are the principal chemotherapeutic drugs for gynecologic malignancies (17). Recent reports showed the potential efficacy of combining AURKA inhibitor with taxanes in epithelial ovarian cancer (18). AURKA has been noted to be a novel therapeutic target for the gynecological malignancies that are particularly resistance to taxanes. However, only a few reports have described a role for AURKA in endometrial cancer. Kurai *et al* found significantly increased expression of AURKA and AURKB in endometrial cancer compared to normal proliferative tissue, with particularly high expression of AURKB in poorly-differentiated endometrial cancer and correlation of this expression with worsening prognosis (19). In a microarray analysis of endometrial cancer tissue, Moreno-Bueno *et al* showed that AURKA is highly expressed in Type II adenocarcinoma (20). Thus, abnormalities in cell cycle checkpoint mechanisms may play a role in carcinogenesis of particular endometrial cancers. However, the significance of its expression in endometrial cancer is not fully understood. The aim of this study was to clarify the significance of AURKA expression in endometrial cancer.

## Materials and methods

### Patients and tissue samples

Tissues were obtained from 162 patients with endometrial carcinoma and from 30 women with normal endometrium who underwent surgery at Keio University (Tokyo, Japan) from 2003 to 2006. All specimens were fixed in 10% phosphate-buffered formalin and embedded in paraffin. Sections of 3 μm were stained with hematoxylin and eosin to confirm the presence of a tumor and to assess the tumor histological characteristics. The items for immunohistochemistry are summarized in Table I. Written informed consent was obtained from the patients regarding use of samples for research. The study was approved by the Ethics Committee of Keio University (approval no. 20130159).

### Immunohistochemical staining

Samples were deparaffinized in xylene and rehydrated in a graded series of ethanol. Antigen retrieval was performed with a 10-min autoclave treatment in 10 mM citrate buffer, pH 7.0. After blocking of endogenous peroxidase activity by dipping sections in 0.3% H_2_O_2_ in PBS for 5 min, sections were incubated overnight with primary antibodies at 4°C in a humid chamber. A primary antibody against AURKA (Trans Genic, Inc., Kumamoto, Japan) was applied at a dilution of 1:200 and anti-Ki67 monoclonal antibody (Dako Denmark A/S, Glostrup, Denmark) was used at a dilution of 1:100. Indirect immunohistochemical staining was performed by the avidin-biotin-peroxidase complex method using a Vectastain Elite ABC kit (Funakoshi Co., Ltd., Tokyo, Japan), using 3,3′-diaminobenzidine as a chromogen and H_2_O_2_. Sections were counterstained with hematoxylin, dehydrated in a graded series of ethanol, dried and coverslipped. TUNEL staining was performed using an *In Situ* Cell Death Detection kit (Roche Diagnostics GmbH, Mannheim, Germany) according to the manufacturer’s instructions.

### Evaluation of immunohistochemical staining

AURKA staining was mainly seen in the nucleus. Overexpression of AURKA was defined as >30% of tumor cells or normal endometrial cells showing nuclear immunoreactivity in five hyper-power fields in each section as previously reported (21). Slides were independently evaluated by two investigators in a blinded manner. In TUNEL and Ki67 staining, positive cells were counted and the percentage of positive cells out of the total number of cancer cells was calculated.

### Cell line and culture

Four human endometrial cancer cell lines were used: SNG-M and HHUA were kindly provided by Professor Shiro Nozwa and Dr Isamu Ishiwata; and HEC-1B and HEC-108 were purchased from the Health Science Research Resources Bank. All cells were maintained in Ham’s F12 (Sigma-Aldrich, St. Louis, MO, USA) supplemented with 10% FBS with appropriate antibiotics at 37°C in a 5% CO_2_ humidified incubator.

### RT-PCR analysis

Total RNA from HEC-108 and -1B, as well as HHUA, and SNG-M cells was extracted for investigation of expression of AURKA using a RNeasy Mini kit (Qiagen, Tokyo, Japan). cDNA was synthesized from 1 μg of total RNA using SuperScript II Reverse Transcriptase (Invitrogen Life Technologies, Carlsbad, CA, USA). AURKA expression was analyzed in a RT-PCR assay using 1 μl of first-stand cDNA as template. AmpliTaq Gold and 10X PCR buffer/MgCl_2_ with dNTP were used in the PCR assay, with analysis using a GeneAMP PCR system 9700 (Applied Biosystems, Foster City, CA, USA). The primer sequences were 5′-ATT GCA GAT TTT GGG TGG T-3′ (sense), and 5′-AAA CTT CAG TAG CAT GTT CCT GTC-3′ (antisense), 472 bp. PCR was performed for 30 cycles (94, 57 and 72°C for 30 sec, respectively).

### Western blot analysis

Western blot analysis was performed to confirm the effect of AURKA inhibition by transfection of AURKA siRNA. siRNA-transfected endometrial cancer-derived cells were rinsed with PBS twice, trypsinized, and centrifuged at 1,000 rpm for 5 min at room temperature. Cells were lysed using a Mammalian Cell Extraction kit (BioVision Research Products, Mountain View, CA, USA). The sample was mixed with sample buffer containing the equivalent volume of 5% β-mercaptoethanol (both from Bio-Rad, Hercules, CA, USA) and the mixture was boiled at 100°C for 5 min. After boiling, the mixture was electrophoresed on a 10% polyacrylamide gel and the proteins were transferred to nitrocellulose membranes (Bio-Rad). The membranes were soaked in PBS containing 1% BSA and 0.1% Tween-20 and incubated at room temperature for 1 h for blocking. They were then reacted with anti-β-actin antibody (1:5,000 diluted, AC-74; Sigma-Aldrich) and anti-AURKA antibody (1:100 diluted; Trans Genic, Inc.) at 4°C overnight, followed by rinsing three times with PBS containing 0.1% Tween (PBS-T) for 10 min each. Anti-β-actin samples were reacted with anti-mouse IgG antibody (PK-6102) and anti-AURKA samples were reacted with anti-rabbit IgG antibody (PK-6101) (both from Vector Laboratories, Burlingame, CA, USA) at room temperature for 1 h. The membranes were rinsed with PBS-T three times and reacted with ABC complex (pre-reacted at 4°C for 30 min, PK-6100; Vector Laboratories) at room temperature for 1 h, then rinsed with PBS-T twice and PBS once, and visualized with DAB (Sigma-Aldrich).

### siRNA AURKA inhibition

siRNA duplexes (siAURKA sense, 5′-AUG CCC UGU CUU ACU GUC ATT-3′; and control sense, 5′-ATC CGC CGC ATA GTA CGT A-3′) were selected and synthesized (Cosmo Bio Co., Ltd., Tokyo, Japan). Transfection of double-stranded siRNA was performed using siFECTOR (B-Bridge International, Inc., Sunnyvale, CA, USA). HEC-1B cells were seeded in 6-cm plates 48 h before transfection. In each plate, 300 pmol of Aurora A siRNA or negative control siRNA and 18 μl of siFECTOR were added to MEM and mixed. After incubation, the siRNA and siFECTOR solutions were mixed gently and added to the plates according to manufacturer’s instructions. The plate was incubated for 48 h until it was ready for further assay.

### Colony formation assay

For colony formation, transfected cells were plated at a density of 1×10^4^ cells/10 cm dish. After 10 days, colonies were stained with 0.1% crystal violet in 50% methanol and photographed with an inverted phase contrast microscope.

### Migration and invasion assay

Migration and invasion assays were performed after transfection of AURKA siRNA for 48 h. BD BioCoat Control Insert Chambers, 24-well plates with an 8-μm pore size (no. 353097) and BD BioCoat Matrigel invasion chambers (no. 354480) (all from BD Biosciences, Franklin Lakes, NJ, USA) were used for migration and invasion assays. In the respective assays, 1 and 2×10^5^ cells/well were plated in the upper compartment in 0.5 ml of serum-free medium. The lower compartment contained 0.75 ml of medium with 10% FCS. Cells were incubated at 37°C in a 5% CO_2_/95% air incubator for 30 h. Cells in the upper compartment were carefully removed with a cotton swab. Cells that migrated or invaded the lower surface of the membrane were fixed and stained using a Diff-Quik kit (Sysmex Corp., Kobe, Japan) and invading cells were counted in five randomly selected microscope fields.

### Chemosensitivity analysis

HEC-1B cells were seeded in 96-well plates for 48 h at 37°C in a 5% CO_2_-humidified incubator. These cells were treated with various concentrations of paclitaxel, cisplatin or adriamycin with or without AURKA siRNA transfection. Viable cells were quantified 48 h after administration of anticancer drugs using a Cell Counting kit (Dojindo, Kumamoto, Japan). Cytotoxicity was measured by determining the IC_50_, the concentration of drug inducing a 50% reduction in cell growth compared with control.

### In vivo experiments

The care and use of animals in the study were approved by the Animal Research Center at Keio University. HEC-1B cells (1×10^7^) were subcutaneously injected at two sites in the flank of female nude mice obtained from CLEA Japan, Inc. (Tokyo, Japan). Twenty days later, mice with tumor formation were randomly divided into five groups treated with control siRNA, AURKA siRNA, paclitaxel, control siRNA and paclitaxel, and AURKA siRNA and paclitaxel. Paclitaxel was administered intraperitoneally at 20 mg/kg and a mixture of AURKA siRNA and atelocollagen (Koken Co., Ltd., Tokyo, Japan) was injected at the tumor site on alternate days. Tumor diameters were measured every 4 days and the tumor volume (mm^3^) was calculated using the following formula: V = length × (wide)^2^ × 1/2. Five tumors were used in each group. Apoptosis was quantified by TUNEL analysis and proliferative index was measured by Ki67 staining.

### Statistical analysis

Correlations between overexpression of AURKA and clinicopathological factors were evaluated by χ^2^ test. The Kaplan-Meier method was used to estimate the probability of disease-free survival (DFS) and a log-rank test was used to compare DFS between groups. P<0.05 was considered to indicate statistical significance. All analyses were performed using IBM SPSS Statistics 21.0 (IBM Japan, Ltd., Tokyo, Japan).

## Results

### Relationship between overexpression of AURKA and clinicopathological factors

**Figure 1:**
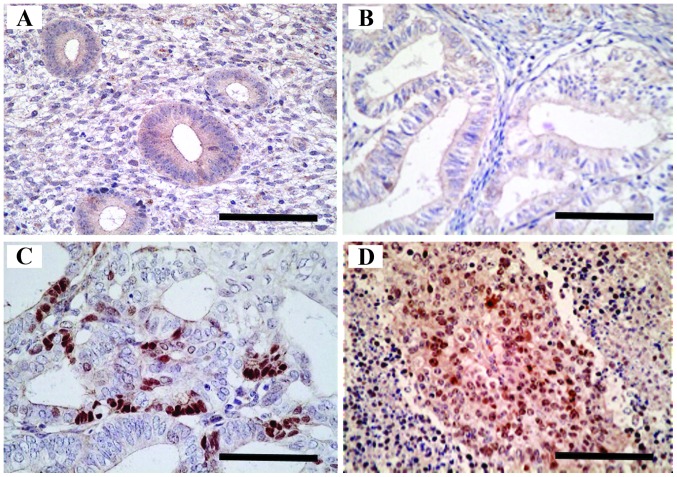
Aurora kinase A (AURKA) expression pattern in normal endometrium and endometrial carcinoma tissues. (A) Normal proliferative phase endometria. (B) Endometrioid adenocarcinoma grade 1. (C) Diffuse positive nuclear staining for AURKA in endometrial adenocarcinoma grade 1. (D) Strong AURKA staining was noted in the nucleus of endometrial adenocarcinoma grade 3. (A) and (B) were negative for overexpression of AURKA. (C) and (D) were assessed as overexpression of AURKA. Scale bars, 100 μm.

AURKA was expressed mainly in the nucleus in normal endometrium and endometrial cancer tissues (Fig. 1A–D). As described in Table I, overexpression of AURKA occurred at a significantly higher rate in endometrial carcinoma than in normal endometrial tissues (49 vs. 3%). Normal endometrial samples that showed overexpression of AURKA were all proliferative phase endometria. In endometrial cancer tissues, overexpression of AURKA was significantly higher in non-endometrioid than in endometrioid adenocarcinoma (81 vs. 47%, P=0.026) and in poorly-differentiated (grade 3) tumors compared with well- (grade 1) or moderately- (grade 2) differentiated tumors in endometrioid adenocarcinoma (70 vs. 41%, P=0.005). However, surgical stage (FIGO 2008) was not associated with overexpression of AURKA (stage I, II vs. III, IV: 47 vs. 57%, P=0.307).

### Association between AURKA expression and patient prognosis

**Figure 2:**
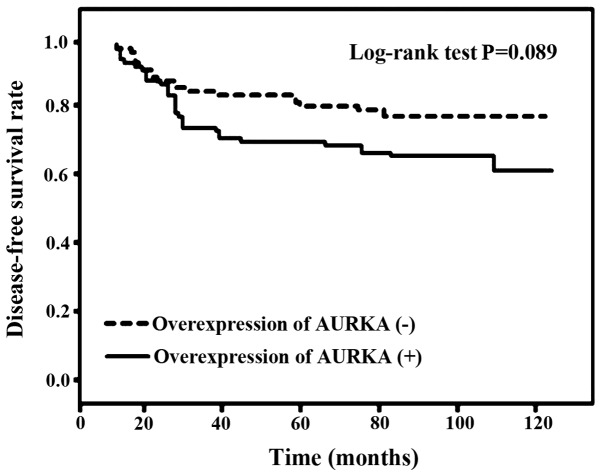
Association between overexpression of Aurora kinase A (AURKA) and patient prognosis. Kaplan-Meier analysis showed the disease-free survival (DFS) rate in relation to overexpression of AURKA. The DFS rate of the patients with overexpression of AURKA was shorter than that of the patients with no overexpression of AURKA tumors.

The follow-up period of the 162 patients ranged from 4 to 127 months, with a median of 86 months. Twelve patients had relapse of the disease and 16 died due to the disease. The recurrence rate tended to be higher (P=0.086; Table I) and DFS tended to be shorter (P=0.087; Fig. 2) in patients with overexpression of AURKA compared to those without overexpression.

### Knockdown of AURKA in HEC-1B cells decreases invasion, migration and colony formation

**Figure 3:**
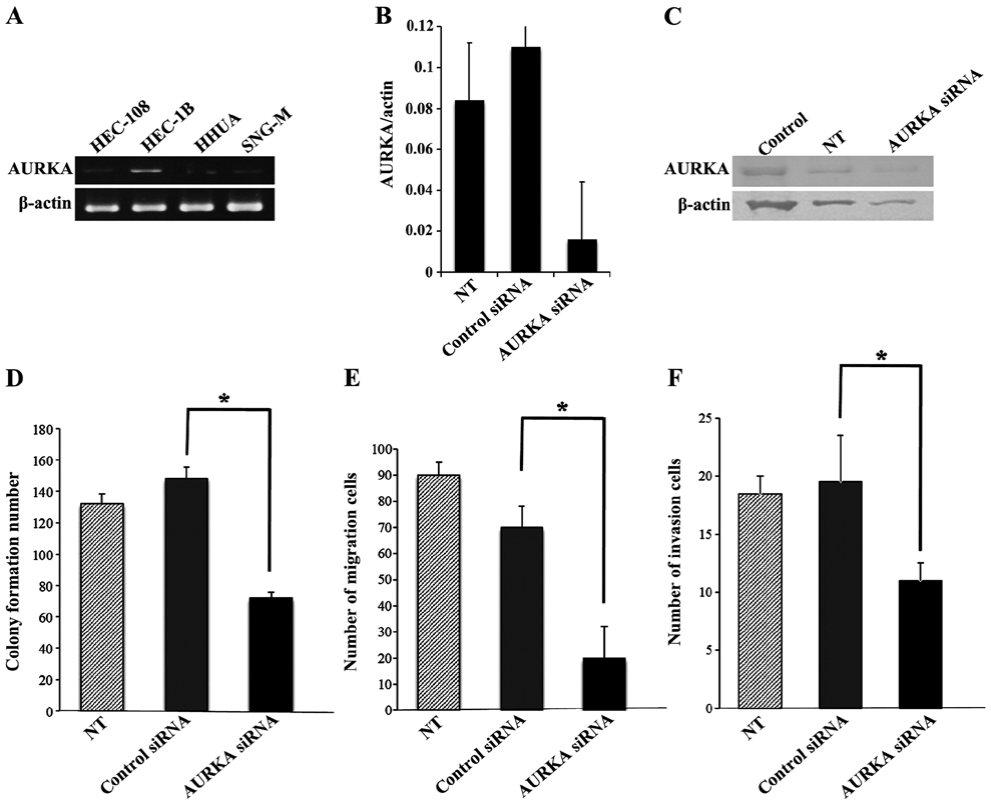
Effects of Aurora kinase A (AURKA) inhibition by transfected siRNA. (A) RT-PCR assay to assess endogenous AURKA expression of HEC-108 and HEC-1B, as well as HHUA, and SNG-M cells. (B) RT-PCR assay of total RNA-extracted AURKA siRNA transfected in HEC-1B cells confirmed AURKA silencing in transfectant. (C) Western blot analysis lysates from AURKA siRNA transfected in HEC-1B cells confirmed AURKA silencing in transfectants. (D–F) Effects of AURKA siRNA transfection on colony formation and invasion and migration in HEC-1B cells. (D) Colony formation assay. (E) Migration assay. (F) Invasion assay. Data are expressed as means ± SE. ^*^P<0.05, n=3.

We screened endogenous AURKA expression in a panel of four endometrial cancer cell lines: HEC-108 (poorly-differentiated), HEC-1B and HHUA (moderately-differentiated), and SNG-M (well-differentiated). RT-PCR showed that HEC-1B cells had a high level of AURKA mRNA expression (Fig. 3A) and these cells were selected for evaluation of the effects of AURKA inhibition or overexpression. To determine whether AURKA has a role in the behavior of endometrial cancer cells, *in vitro* experiments were performed to analyze the effects of AURKA loss of function on cell proliferation, invasion and migration. siRNA-targeting AURKA produced efficient knockdown of AURKA in HEC-1B cells, as shown by RT-PCR and western blot analysis: the AURKA mRNA level decreased to 13% of that with scrambled control siRNA (Fig. 3B) and the AURKA protein level also decreased (Fig. 3C). Transfection with AURKA siRNAs significantly decreased cell proliferation, invasion and migration of HEC-1B cells, compared with transfection of control siRNA (Fig. 3D–F).

### AURKA knockdown by siRNA enhances the chemosensitivity of HEC-1B cells to paclitaxel

**Figure 4:**
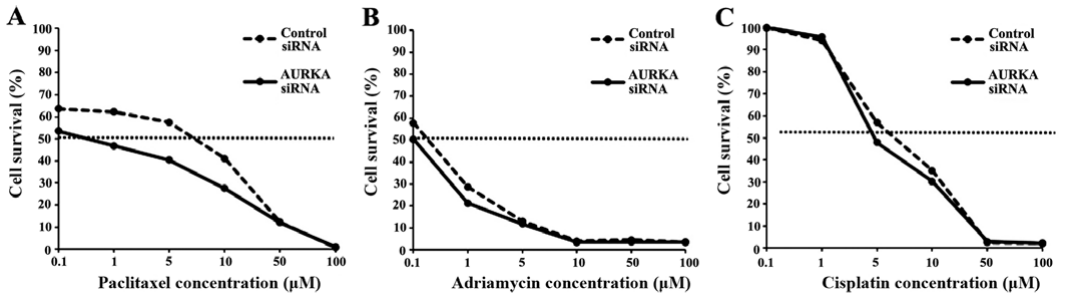
Effects of Aurora kinase A (AURKA) siRNA transfection on chemosensitivity in HEC-1B cells. (A–C) HEC-1B cells were treated with various concentrations of paclitaxel, cisplatin or adriamycin with or without AURKA siRNA transfection. Percent survival was determined 48 h after administration of anticancer drugs using a Cell Counting kit.

Recent reports showed that the upregulation of AURKA contributes to chemoresistance in a human cell line, pancreatic esophageal, breast and colon carcinoma cells (22–26). To determine whether AURKA knockdown had an effect on chemosensitivity, a cytotoxicity assay was performed to measure IC_50_ values of paclitaxel, adriamycin, and cisplatin, which are widely used in gynecological cancer chemotherapy, before and after AURKA knockdown in HEC-1B cells. Only the IC_50_ for paclitaxel changed after AURKA knockdown (Fig. 4, Table II), indicating that AURKA expression is correlated with sensitivity to paclitaxel. These results suggest that AURKA siRNA and paclitaxel in combination may be effective for treatment of endometrial cancer.

### AURKA siRNA and paclitaxel in combination enhances chemosensitivity in vivo

**Figure 5:**
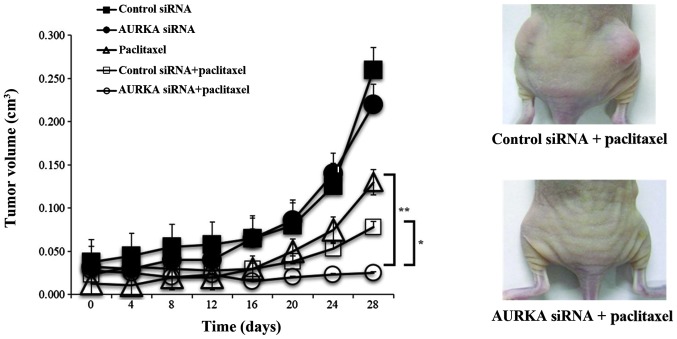
Combination effects of Aurora kinase A (AURKA) siRNA and paclitaxel in HEC-1B xenograft model. HEC-1B cells (1×10^7^) were subcutaneously injected in the flank of female nude mice. Twenty days later, mice with tumors were randomly divided into five groups treated with control siRNA, AURKA siRNA, paclitaxel, control siRNA and paclitaxel, and AURKA siRNA and paclitaxel. Paclitaxel was administered intraperitoneally at 20 mg/kg and a mixture of AURKA siRNA and atelocollagen was injected at the tumor site on alternate days. Tumor diameter was measured every 4 days, and mean tumor volumes were plotted against days of treatment. Scale bars, SD. ^*^P<0.1, ^**^P<0.05.

**Figure 6:**
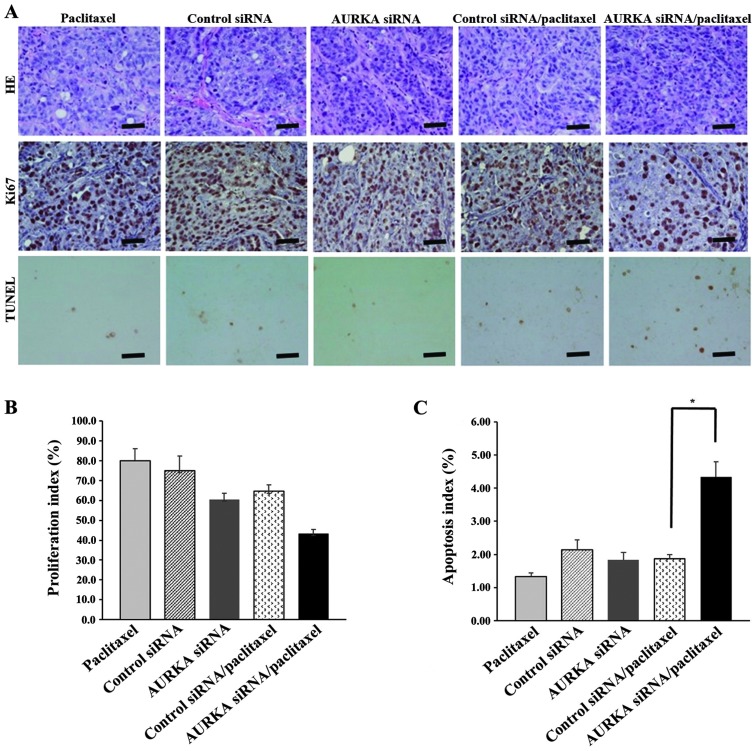
Pathological findings. (A) H&E staining and TUNEL and Ki67 staining of HEC-1B tumor sections after indicated treatment. (B) Ki67 proliferative index. (C) Apoptosis index. Scale bars, 50 μm. Ki67 and TUNEL staining, positive cells were counted and the percentage of positive cells out of the total number of cancer cells was calculated. Data are expressed as means ± SE. ^*^P<0.05, n=5.

We analyzed antitumor activity in nude mice bearing established HEC-1B tumors using treatment with control siRNA, AURKA siRNA, paclitaxel, control siRNA and paclitaxel, and AURKA siRNA and paclitaxel. In comparison with other treatments, the combination of AURKA siRNA and paclitaxel showed a tendency to inhibit tumor growth (P<0.1; Fig. 5). Immunohistochemical analysis of apoptosis (TUNEL analysis) and proliferation (Ki67) after treatment (on day 28) showed that the combination of AURKA siRNA and paclitaxel significantly increased the number of TUNEL-positive cells (P<0.05) and showed a trend for decreasing the number of Ki67-positive cells (Fig. 6A–C). Therefore, these *in vivo* data indicate enhanced antitumor activity of AURKA siRNA and paclitaxel combination in endometrial cancer.

## Discussion

AURKA, also called STK15, is a serine/threonine kinase that maintains cell division by regulating centrosome separation, bipolar spindle assemble, and chromosome segregation (5,6,27). AURKA is also linked to the processes of G2-M arrest, apoptosis and ectopic expression leading to bypass of G2-M in the DNA damage-activated checkpoint system (9,28). Aberrant amplification of AURKA occurs in human malignancies such as breast (27), pancreatic (12,29), colorectal (10), gastric (13), and ovarian carcinomas (27,30) and in some cases is associated with a poor prognosis (31–33). Although AURKA is a potential new oncogenic target, the role of this protein in endometrial cancer is unclear.

In this study, we found an association of overexpression of AURKA with clinicopathological factors in endometrial cancer. Immunohistochemistry showed overexpression of AURKA in endometrial cancer tissues compared with normal endometrium, indicating that upregulation of AURKA is a frequent abnormality in endometrial cancer. Overexpression of AURKA was associated with tumor grade and histological type in endometrial cancer tissues in χ^2^ tests and a tendency for this association remained in logistic regression analysis, but this was not significant (data not shown). Patients with overexpression of AURKA also tended to have shorter DFS and a higher recurrence rate. These results suggest that elevated AURKA tumor expression may be an indicator of rapid cell division, rather than the cause of a malignant phenotype.

Correlation of overexpression of AURKA with malignant phenotypes has been shown in several cancers (11,12,34,35). Amplification of AURKA causes chromosomal instability (6) that may help tumor cells acquire invasive and metastatic phenotypes. Our results showed that specific inhibition of AURKA by siRNA suppressed endometrial cancer cell growth, migration and invasion. The mechanisms through which AURKA influences cell migration and invasion are not completely defined, but previous studies suggest roles for p53 (8,36,37), RAS (38), AKT (39,40), and MAPK (41). The level of p53 protein, which is a key player in this checkpoint, is increased in AURKA-overexpressing cells and apoptosis is inhibited by deletion of p53. Given that malignant tumor formation does not occur in a mouse model with AURKA overexpression after a long latency period, additional factors such as p53 inactivation and expression of pro-survival proteins are likely to be required for tumorigenesis (4). This hypothesis is supported by the clinical observation that AURKA overexpression is correlated with p53 mutation in hepatocellular carcinomas, and that tumors with both AURKA overexpression and p53 mutation have a worse prognosis than those with p53 mutation alone (42). In this study we showed that overexpression of AURKA was significantly higher in non-endometrioid adenocarcinoma and in grade 3 tumors that are classified Type II tumors. From these findings, we speculated that overexpression of AURKA may be associated with p53 mutation, and caused poor prognosis in Type II tumors.

Several reports have shown that upregulation of AURKA results in resistance to anticancer agents including paclitaxel (18,22,43,44), and docetaxel (23,44) in various cancers. Our *in vitro* data showed that AURKA expression was correlated with sensitivity to paclitaxel and our *in vivo* results suggested that paclitaxel and AURKA siRNA in combination had significantly enhanced antitumor efficacy. Taxanes such as paclitaxel bind to microtubules and inhibit dissociation of tubulin subunits. This inhibition of microtubule depolymerization by paclitaxel in tumor cells prevents reconstruction of microtubules and formation of the spindle, generating aberrant cell division. The spindle formation checkpoint recognizes this abnormality and triggers apoptosis of the tumor cell, causing the tumor to shrink (7,45,46). Overexpression of Aurora kinases causes dysfunction of checkpoints in cell division and permits the cell to enter anaphase in an improper state (22). Thus, in the presence of overexpressed Aurora kinases, taxane-based anticancer agents cannot induce apoptosis of aberrant cells and have reduced sensitivity. Conversely, drugs that inhibit Aurora kinases may suppress resistance to apoptosis induced by taxanes and enhance antitumor action. For this reason, several small-molecule Aurora kinase inhibitors have been developed that exhibit preclinical activity against various solid tumors. These include MLN8237, Hesperadin, VX-680, VE465 and Barasertib, and clinical trials are ongoing to verify the effects of these inhibitors (47). Combination therapy of paclitaxel and AURKA inhibition using siRNA or an AURKA inhibitor may also allow reduction of the dose of paclitaxel, with the result of fewer side-effects.

In summary, our data on endometrial carcinoma show that overexpression of AURKA is strongly associated with tumor grade and histological type, and that there is a correlation between expression of AURKA and sensitivity to paclitaxel. These results suggest that AURKA may be a biomarker for identification of a subgroup of patients with resistance to treatment and a poor prognosis, and a promising target for novel therapeutics for endometrial cancer. Combination treatment using AURKA inhibitors and paclitaxel may be particularly effective for cases of endometrial cancer that are resistant to conventional treatment.

## Figures and Tables

**Table I:** Relationship between overexpression of AURKA and clinicopathological factors.

Overexpression of AURKA	Negative (n=110)	Positive (n=82)	aP
Endometrial tissues (NEM vs. EC)			0.000
NEM			
Proliferative phase	13	2	
Secretory phase	15	0	
EC	82	80	
FIGO surgical stage (I, II vs. III, IV)			0.307
I	63	53	
II	3	6	
III	14	18	
IV	2	3	
Histological type (Non-EA vs. EA)			0.026
Non-EA			
Serous adenocarcinoma	2	4	
Clear cell adenocarcinoma	0	5	
EA	80	71	
Grade (G1 and 2 vs. G3)			0.005
G1	48	34	
G2	23	16	
G3	9	21	
Recurrence rate (%)	19.5	31.25	0.086

a χ^2^ test.

AURKA, Aurora kinase A; NEM, normal endometrium; EC, endometrial cancer; Non-EA, non-endometrioid adenocarcinoma; EA, endometrioid adenocarcinoma.

**Table II:** IC_50_ in HEC-1B cells with or without AURKA siRNA.

	IC_50_ (μM)	
	
	Control siRNA	AURKA siRNA
Paclitaxel	2.0	40.0
Adriamycin	0.20	0.10
Cisplatin	7.0	6.0

AURKA, Aurora kinase A.
